# Revealing Historic Invasion Patterns and Potential Invasion Sites for Two Non-Native Plant Species

**DOI:** 10.1371/journal.pone.0001635

**Published:** 2008-02-20

**Authors:** Jacob N. Barney, Thomas H. Whitlow, Arthur J. Lembo

**Affiliations:** 1 Department of Plant Sciences, University of California Davis, Davis, California, United States of America; 2 Department of Horticulture, Cornell University, Ithaca, New York, United States of America; 3 Department of Crop and Soil Sciences, Cornell University, Ithaca, New York, United States of America; University of Zurich, Switzerland

## Abstract

The historical spatio-temporal distribution of invasive species is rarely documented, hampering efforts to understand invasion dynamics, especially at regional scales. Reconstructing historical invasions through use of herbarium records combined with spatial trend analysis and modeling can elucidate spreading patterns and identify susceptible habitats before invasion occurs. Two perennial species were chosen to contrast historic and potential phytogeographies: Japanese knotweed (*Polygonum cuspidatum*), introduced intentionally across the US; and mugwort (*Artemisia vulgaris*), introduced largely accidentally to coastal areas. Spatial analysis revealed that early in the invasion, both species have a stochastic distribution across the contiguous US, but east of the 90^th^ meridian, which approximates the Mississippi River, quickly spread to adjacent counties in subsequent decades. In contrast, in locations west of the 90^th^ meridian, many populations never spread outside the founding county, probably a result of encountering unfavorable environmental conditions. Regression analysis using variables categorized as environmental or anthropogenic accounted for 24% (Japanese knotweed) and 30% (mugwort) of the variation in the current distribution of each species. Results show very few counties with high habitat suitability (≥80%) remain un-invaded (5 for Japanese knotweed and 6 for mugwort), suggesting these perennials are reaching the limits of large-scale expansion. Despite differences in initial introduction loci and pathways, Japanese knotweed and mugwort demonstrate similar historic patterns of spread and show declining rates of regional expansion. Invasion mitigation efforts should be concentrated on areas identified as highly susceptible that border invaded regions, as both species demonstrate secondary expansion from introduction loci.

## Introduction

The economic and environmental damage that invasive species are wreaking on our native landscapes continues to grow [1], [2]. Much of this is a consequence of not recognizing an invasion until the non-native species is naturalized locally, and spreading at larger spatial scales (i.e., landscape or regional scales). Therefore, great effort has gone into identifying the potential range of an invasive species before it has spread at large spatial scales. These modeling approaches include ecological niche modeling (e.g., [3]), climate-matching analyses (e.g., [4]), and habitat suitability prediction (e.g., [5]). Many studies require information on the distribution of the species in its native range and regions where it has been introduced, or the species’ responses to climatic conditions of the target region. This information is typically entirely unknown or difficult to estimate with any degree of accuracy, thereby precluding accurate identification of invadable habitat. Without a synthetic framework from which to build a robust predictive model for non-native species, many researchers are beginning to focus on using existing spatial distributions to make predictions of sites with suitable habitat (i.e., invadable areas) at various spatial scales [5], [6], [7], [8].

Habitat suitability can be adequately assessed in the target region based on knowledge of successful (present) and unsuccessful (absent) introduction events. For example, Morisette *et al.* [5] compiled 45 datasets, composed of >32,000 presence/absence locations, for tamarisk (*Tamarix* spp.**)** in the contiguous US. With this rich dataset they were able to produce a high-resolution map showing habitat suitability based only on information in the introduced range. However, datasets of this scale are rare, especially for an entire country, require vast resources to compile, and to be useful (as with most models), require the assumption that absence of evidence equates to evidence of absence. Extrapolation is exaggerated when using point data for areas of low resolution: for example, the entire state of Texas was modeled using 64 points, while Colorado (∼one-third as large) was modeled using >7,000 data points [5]. These studies also ignore dispersal mechanisms and corridors, and any spatial autocorrelation that might exist in the distribution [9]. Therefore, a need exists for models that incorporate the current distribution of an invasive species with knowledge of introduction sites and dispersal pathways. This approach can be accomplished by incorporating detailed historical information at large spatial scales [10], [11], [12], [13]. Combining coarse-resolution distribution patterns (i.e., non-point based presence/absence data) with population biology and information on dispersal patterns and mechanisms results in a multi-scalar understanding of the invasion process, and yields information on un-invaded regions with suitable habitat that can be protected [14].

Historical re-creations of species invasions have the benefit of elucidating sites of early successful (and unsuccessful) introductions, locations of suitable habitats at various spatial scales, modes of introduction, and dispersal pathways. For plant species, herbarium records are most often used to study historic phytogeography [12], [15], [16], [17], [18]. Herbarium records contain information on the location of the population, collection date, as well as a dried specimen from the population that can be used for taxon verification and molecular ecology studies. Herbarium data can be applied from local to regional spatial scales across chosen timeframes, allowing descriptions of local processes and larger mechanisms of range expansion and habitat suitability [14]. Combining this historical information with a descriptive-predictive statistical model can lead to a better understanding of invasive species establishment, dispersal, and habitat suitability.

The objectives of this study were to investigate the historic and potential phytogeography at the US county level for the invasive perennial weeds Japanese knotweed (*Polygonum cuspidatum* Sieb. & Zucc.) and mugwort (*Artemisia vulgaris* L.) using herbarium data. Both Japanese knotweed and mugwort are perennial geophytes with long histories of human use, but contrasting primary introduction pathways to North America [12]. Japanese knotweed was introduced intentionally via ornamental plantings to >88 counties across the contiguous US since the 1870s, while mugwort was introduced accidentally to shipping ports at least 17 times, largely in the Northeastern US [12]. Despite the differences in geography and number of known introduction loci, Japanese knotweed and mugwort have similar current distributions. Therefore, we were interested in comparing the historic patterns of invasion, and modeling the potential distribution to identify sites with suitable habitat for invasion. More specifically, we wanted to address the following questions: 1) Do historical patterns of invasion at the county level differ between Japanese knotweed and mugwort?; 2) Using environmental and anthropogenic variables can we create a habitat suitability map at the resolution of US counties?; and 3) How many locations provide suitable habitats for future invasion–or how close are these invaders to a coarse-scale carrying capacity?

## Results

### Spatio-temporal autocorrelation analysis

#### Japanese knotweed

**Figure 1:**
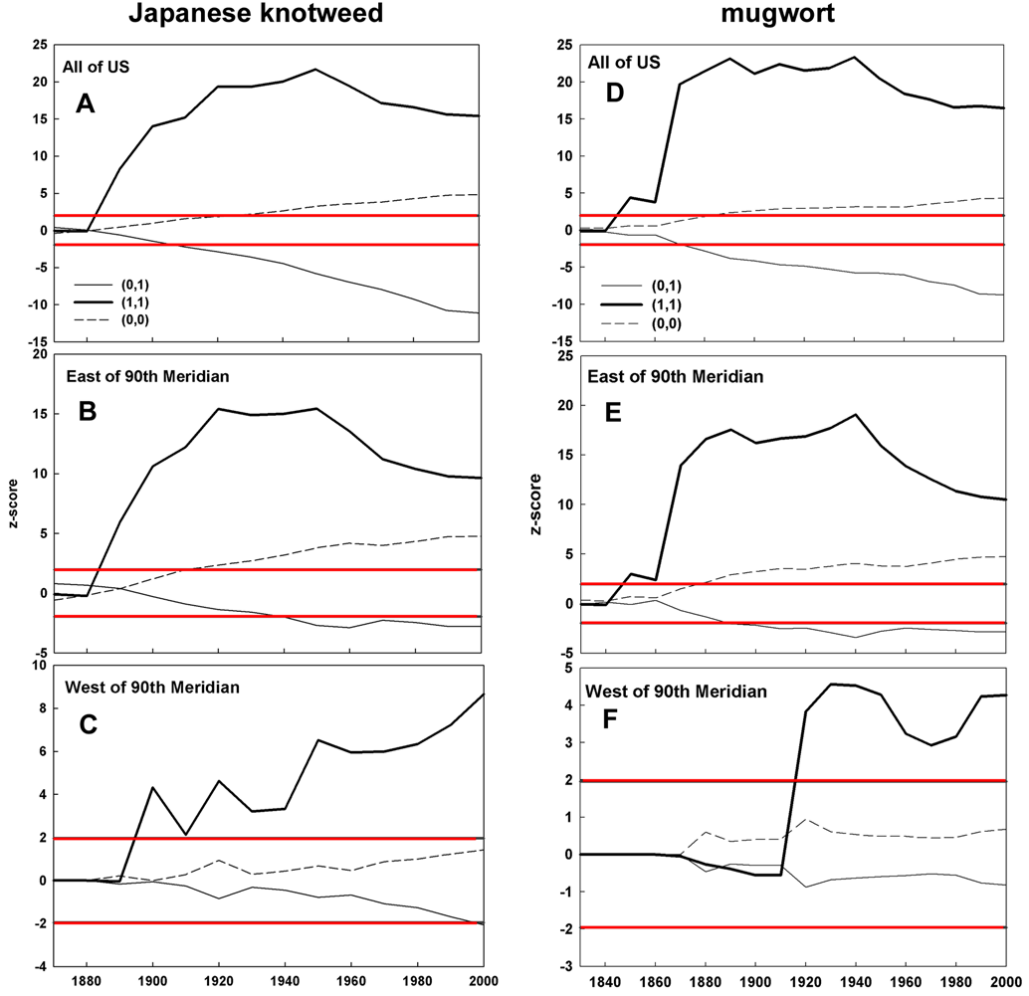
Spatial clustering of invaded counties throughout the invasion history. Join count z-score test statistic values at each decadal interval since initial introduction for the contiguous US (A,D), and east (B,E) and west (C,F) of the 90^th^ meridian for Japanese knotweed (left column) and mugwort (right column). The bold line (1,1) represents adjoining counties both with a Japanese knotweed or mugwort record, the solid line (0,1) represents adjoining counties where one county has a weed record and the other does not, and the dashed line (0,0) represents adjoining counties where neither contain a weed record. Horizontal red bars show positive and negative Z-score thresholds at *P* = 0.05.

Counties with a Japanese knotweed record were spatially distributed as would be expected from a random distribution (i.e., no spatial autocorrelation) for the first two decades after introduction (Figure 1a). From 1890 to 1910, counties with Japanese knotweed were more clustered, but adjacent present-absent counties were not different than would be expected from a random distribution (0,1 in Figure 1a). From 1920 to 2000, adjacent present-present counties were greater than would be expected from a random distribution, and adjacent present-absent counties were fewer than expected. A similar pattern was observed east of the 90^th^ meridian, with counties with Japanese knotweed clustering in 1890 and onward, and adjacent present-absent counties being fewer than expected from a random distribution in 1950 and onward (Figure 1b). However, clustering of counties with Japanese knotweed did not begin until 1900 west of the 90^th^ meridian, while adjacent present-absent counties were never fewer than expected from a random distribution (Figure 1c).

#### Mugwort

For the first three decades following initial introduction in the 1820s, the spatial distribution of mugwort was randomly distributed (Figure 1d). In the 1850s and onward, counties with a mugwort record were more aggregated than would be expected from a random distribution (1,1 in Figure 1d). Additionally, there were fewer adjacent present-absent counties than would be expected from a random distribution (0,1 in Figure 1d). The same pattern was seen for counties east of the 90^th^ meridian (Figure 1e). However, west of the 90^th^ meridian, the spatial distribution of counties with mugwort was no different than that expected for 100 years (1820–1920, Figure 1f). From 1920 to 2000, counties in the west with a mugwort record were clustered. However, adjacent present-absent counties were distributed as would be expected from a random distribution.

### Habitat suitability analysis

#### Japanese knotweed

Of the 11 possible variables (county area not included as it was a covariate), five were significantly correlated with Japanese knotweed distribution and explained 24% of the variation (Tables 1 and 2). Three predictors were in the anthropogenic category, and two in the environmental category, with minimum temperature and county human population being most strongly correlated (highest |b*|). Two predictors were negatively correlated, while three were positively correlated with Japanese knotweed distribution. The resulting model was 30% accurate in predicting counties with suitable habitat where a population existed (Table 2). The model predicts five additional counties with suitable Japanese knotweed habitat at >80% probability, which are not currently invaded (Table 2).

**Table 1:** Logistic regression parameters for Japanese knotweed and mugwort.

Variable	Japanese knotweed			mugwort		
	b	b*	|b*|	b	b*	|b*|
County area†	−1.01	−0.11	0.11	−0.97	−0.11	0.11
Minimum temperature	−0.26	−0.35	0.35	−0.29	−0.40	0.40
Population†	1.85	0.34	0.34	1.88	0.35	0.35
Mean precipitation†	4.90	0.27	0.27	3.70	0.21	0.21
Proportion urban population	−1.01	−0.09	0.09	−0.97	−0.08	0.08
Highway length	0.002	0.09	0.09	0.002	0.11	0.11
Mean elevation†	-	-	-	−0.71	−0.13	0.13
Proportion area agriculture	-	-	-	−1.85	−0.14	0.14

The parameters b and b* are the regression and standardized regression coefficients [30], respectively, and |b*| is the absolute value of the standardized regression coefficient.

Variables with a dagger (†) are log_10_ transformed.

**Table 2:** Logistic regression model accuracy and efficiency.

	Japanese knotweed		mugwort	
	Training dataset	Testing dataset	Training dataset	Testing dataset
*Accuracy*				
Observed	389	202	284	146
Expected	133	61	96	43
% correct	34	30	34	29
*Efficiency*				
False positives	57	23	43	23
False negatives	256	141	188	103
Un-invaded: ≥80%†	5		6	
R^2^	0.24		0.30	

Observed and expected number of US counties where each invasive was present for habitat suitability ≥0.5 for both the training and test datasets.

† These values are for total number of counties without a population but have a probability ≥80% based on logistic regression results.

**Figure 2:**
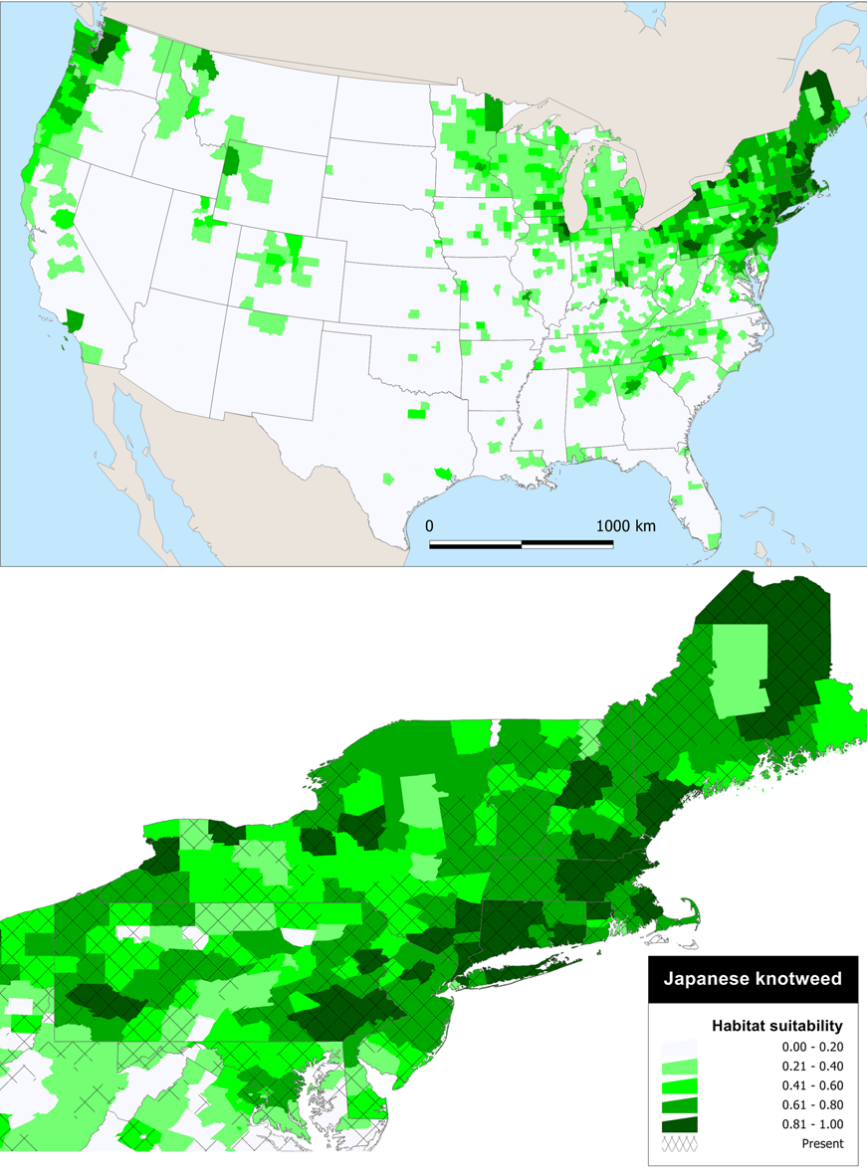
Habitat suitability of Japanese knotweed. Model predictions of habitat suitability based on the environmental and anthropogenic factors in Table 1.

The spatial distribution of counties with high probabilities of Japanese knotweed is aggregated in the Northeast, Great Lakes region, and Pacific Northwest (Figure 2). Appalachia, from Alabama to Pennsylvania, also has moderately high probabilities of a Japanese knotweed invasion. However, the interior of the contiguous US has a very low (<25%) habitat suitability (Figure 2). Interestingly, despite several dozen counties in the Mid-Atlantic and southeastern Midwest with a Japanese knotweed population, the habitat suitability is very low.

#### Mugwort

The same five variables were significantly correlated with mugwort distribution as were with Japanese knotweed, with the addition of elevation and proportion agriculture (Table 1). Minimum temperature and county human population were most strongly correlated, though opposite in sign (negatively vs. positively respectively).

**Figure 3:**
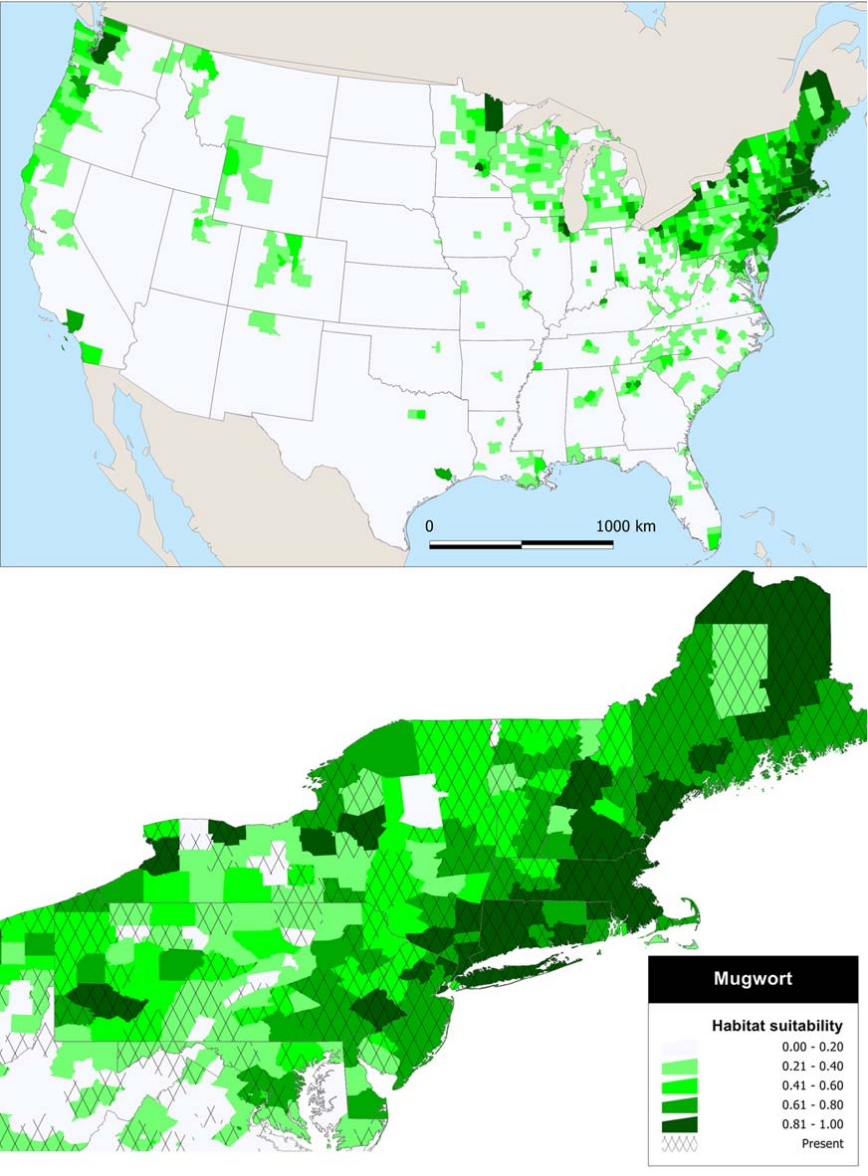
Habitat suitability of mugwort. Model predictions of habitat suitability based on the environmental and anthropogenic factors in Table 1.

The spatial distribution of counties with high habitat suitability occur primarily in the Northeast, the Great Lakes region, and the Pacific Northwest (Figure 3). As with Japanese knotweed, many counties in the Mid-Atlantic States have documented populations, but the model shows very low habitat suitability.

## Discussion

Baker [19] states that any species introduced to a novel habitat (i.e., one where it is not native) can be described by geographically disparate founding populations early in the invasion, followed by expansion from these source loci, as opposed to a stochastic filling of the same space. We found this pattern of an advancing invasion front in both Japanese knotweed and mugwort in the contiguous US, with a lag phase–here defined as a regionally stochastic distribution–of 20–40 years after earliest herbarium record. Both species are rhizomatous perennials that recruit locally via vegetative propagules, but can disperse regionally via seeds [20], [21]. Therefore, the regional dispersal lag phases of 2–4 decades are longer than would be expected for an anemochorous annual [14], [22]. However, west of the 90^th^ meridian the lag phase was around 100 years for mugwort, likely reflecting the fewer introduction events to this area [12], larger average size of the counties, and unsuitable environmental conditions as exemplified in the model output. This suggests that the idea of lag phases should be operationally defined in a spatio-temporal context to alleviate issues of scale, propagule pressure, and non-uniformity of introduced habitats.

The combination of many present-present joins and few present-absent joins after the lag phase for each species suggests that counties with a herbarium record are in large adjoining groups, versus many small clusters, which suggests an advancing invasion front at the regional scale. We cannot determine if the populations identified in adjacent counties resulted from the same source population (i.e., documented sites of introduction) without genetic analysis (e.g., [23]). However, our results strongly support secondary dispersal from introduction loci for both species despite their dissimilar introduction histories and geographies, due to their primarily vegetative recruitment. Japanese knotweed was intentionally introduced to at least 88 sites in the US, while mugwort was introduced to at least 34 sites [12]. Mugwort was frequently introduced accidentally via ship ballast (17 times to 6 ports), and untold numbers of landscapes throughout the US in contaminated nursery stock [21]. Therefore, large-scale expansion can be achieved quickly (<200 years) from relatively few introduction sites, regardless of the introduction vector.

The logistic regression model using the current distribution of mugwort and Japanese knotweed at the US county spatial scale performed fairly well, describing 24–30% of the variation. Both environmental and anthropogenic variables were important in describing suitable habitat. However, only 30% accuracy was achieved in the test datasets. Morisette et al. [5] using a similar method, but at the resolution of individual tamarisk trees, achieved >90% accuracy. Their high accuracy could be a result of the resolution of their study combined with the “habitat” variables used, which included remotely sensed MODIS data. It is also likely that the spatial resolution of US counties, which ranged from 5 to >52000 km^2^, may be too coarse to adequately represent environmental conditions within geopolitical boundaries, especially in the western US where counties are orders of magnitude larger than those in the east. Additionally, the low model accuracy at this spatial scale suggests that other variables not considered here also play important roles in the phytogeographic distribution of these species (e.g., propagule pressure, local disturbances, the nursery trade). Nevertheless, our model was able to capture regional trends in the distribution of each species, and incorporate variables other than just climate (e.g., [4]), which undoubtedly influence distribution (e.g., highway length as a metric for anthropochory).

Despite introductions across most of the contiguous US, the Northeast and Pacific Northwest–regions characterized by wet warm springs and moderate summers–offer the highest habitat suitability for both perennials. The variable most strongly correlated with Japanese knotweed and mugwort distributions was minimum temperature. Japanese knotweed is particularly sensitive to frost, thereby limiting range expansion into areas that experience early or late-season frosts (e.g., high elevation or latitude) [20]. Surprisingly, neither species seems to thrive at southerly latitudes (high minimum temperatures), despite introductions south of 36°N and native ranges that include semi-tropical locales. This could reflect insufficient time to establish in these locations, or a lack of environmental tolerance for high minimum temperatures or some other (negative) environmental correlate occurring at these latitudes [3]. Precipitation is also strongly positively correlated with the distribution of each species, especially Japanese knotweed, which favors moist soils [20]. These variables, and their relative explanatory power, give a broad representation of the regional environmental tolerance and anthropogenic influence for each species.

Many of the counties that currently have a mugwort or Japanese knotweed population, but possess low habitat suitability, are typically isolated and not bordered by other invaded counties. This pattern is especially clear west of the 90^th^ meridian, where most populations do not expand beyond the boundaries of the county of introduction. This likely reflects the cultivation of these western introductions [12], as people moved plants with them as they migrated west. Often these new climates were unsuitable, and cultivation was necessary to maintain a viable population [24], [25]. The habitat suitability model corroborates this hypothesis by showing low suitability in regions where Japanese knotweed and mugwort are minor or benign introductions (e.g., the southwest and Mid-Atlantic), and occur in isolation.

A very small number of US counties remain un-invaded that have high habitat suitability, and all occur in the Northeast and Pacific Northwest–regions where both species are problematic and occur at high densities locally [20], [21]–and are often surrounded by invaded counties (Figures 2 and 3). These “invasion holes” are likely the result of a lack of local collectors or collecting institutions, populations occurring in inaccessible locations, or occurring in low abundances [18], [26]. Conversely, if counties with high habitat suitability do not currently support a population, they should be considered high priority areas for monitoring. Waterways and roads into these counties, especially from corridors where a population exists “upstream” or “down the road”, should be monitored as Japanese knotweed and mugwort effectively disperse along these corridors [12]. This implies that in ∼200 years since the initial introduction both species have nearly reached the limits of invasion at the regional scale. Of course this passes no judgment on local or population scales, which will undoubtedly continue to expand. The hypothesis of these species reaching the extent of the fundamental niche (i.e., sites of suitable habitat without considering biotic interactions) in the contiguous US, could be corroborated with climate-matching (e.g., [4]) and niche-modeling [3], which incorporate information on native population environmental tolerance.

We acknowledge the limitations involved with using herbarium records as a proxy for current species distributions [18], [26]. Counties also have limitations when used in a spatial analysis as they are geopolitical artifacts and do not delimit environmental boundaries. However, by assessing spatial patterns at the county level we are circumventing some of the biases found in studies based on point data (e.g., [5], [8]). Additionally, the spatial and temporal scale at which we conducted our analyses precluded all other data sources. Benefits of using coarser spatial scales are the higher probability of species detection (i.e., larger areas are surveyed), the relative ease of obtaining the data versus that necessary for point-data [e.g., 5], and the opportunities to derive estimates of habitat suitability at regional scales. If large-scale trends can be determined from datasets that are more widely available perhaps a more robust model can be developed to determine species invasiveness, habitat invasibility, and future invadable sites [14].

This study demonstrated that coarse-scale distributions based on herbarium data could be used to elucidate historical distribution dynamics, introduction and dispersal patterns, and estimate regional carrying capacity. The large-scale dispersal of Japanese knotweed and mugwort, invasive species with contrasting introduction histories in the US, could be explained by secondary dispersal from primary introduction sites into adjacent counties. However, when Japanese knotweed and mugwort were introduced into sites which were not favorable, for example the eastern Midwest, they did not spread regionally. Both environmental and anthropogenic variables described the habitat suitability of both species, with minimum temperature and human population being the most strongly correlated. In less than 200 years since initial introduction, very few un-invaded counties remain with high habitat suitability for either species–suggesting Japanese knotweed and mugwort are approaching their regional carrying capacity in the contiguous US. Analyses at the larger spatial scale, combined with knowledge on population biology will aid in resolving a more complete picture of the invasion process and could aid land managers in arresting future range expansion and mitigate current impacts. A major challenge for management, however, will be incorporating the unknown and potentially unpredictable impact of global climate change on the potential spread of established invaders. Future research should strive to integrate climate change models into dynamic models predicting range expansions.

## Methods

### Phytogeographic data

US counties were chosen for the analysis unit as species presence/absence information is more likely to be available for counties, and our objective was to investigate coarse-scale patterns and processes. For the purposes of this study we confined our analysis to the continental US due to the much larger average size of Canadian municipalities, vast areas of Canada that are uninhabited, and lack of available spatial data for Canada. Phytogeographic information on mugwort and Japanese knotweed were obtained from herbarium records (see [12] for complete information on data). Herbarium records from 249 institutions were obtained and verified for correct identification. A total of 1476 Japanese knotweed records representing 577 US counties, and 1277 mugwort records representing 432 US counties were obtained. For analysis purposes the earliest record per county was used [13], [16].

### Spatio-temporal autocorrelation analysis

The current distribution of a species can result either from many independent introductions that spread only locally (a stochastic distribution), or regional secondary spread from initial introduction sites [27]. At larger spatial scales, in our case US counties, we would expect counties with invasive populations to be randomly distributed early in the invasion, and become more clustered as the invasion spreads. If the invader reaches a favorable site, and is able to spread outside its introduced location(s), we would expect it to first move to adjacent counties. Therefore, based on the number of counties reporting a population, and the spatial arrangement of these counties, we can calculate the statistical likelihood of these being clustered versus randomly distributed–spatial autocorrelation. The join count analysis calculates a Z-score test statistic that compares the observed number of adjoining counties with an invasive population to the expected number assuming a random distribution [28]. The expected value for each join count is given by
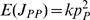


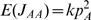


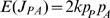
where J_PP_, J_AA_, and J_PA_ represents the present-present, absent-absent, and present-absent county joins respectively, k represents the total number of joins on the map, and p_P_ is the probability of a county having an invasive population and p_A_ is the probability of a county not having an invasive population. The expected standard deviation for each join are given by
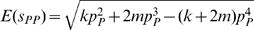


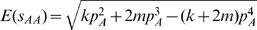


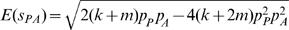
where k, p_P_, and p_A_ are as before, and m is defined as
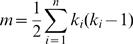
where k_i_ is the number of joins in the *i*th area. Spatial Query Language (SQL) commands written in the geographic information system (GIS) software package Manifold v6.5 (Manifold Net Ltd., Carson City, NV, USA) were used to calculate k and m. We then calculated the Z-score test statistics, which are defined as
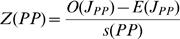


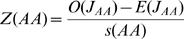


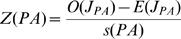
where O(J_PP_), O(J_AA_), and O(J_PA_) are the observed number of present-present, absent-absent, and present-absent county joins respectively. Z(PP) >1.96 (determined at α = 0.05) suggests that counties with an invasive population are more clustered than would be expected from a random distribution. Z(PA) <−1.96 suggests there are fewer adjoining counties with and without a population than would be expected from a random distribution (i.e., invaded counties are clustered).

The observed and expected number of joins, their standard deviations, and their Z-score test statistics for both mugwort and Japanese knotweed were calculated at each decadal interval since initial introduction for the contiguous US. We also divided the US along the 90^th^ meridian into East and West regions, which approximates the Mississippi River, and ran identical join count analyses on each section individually. This was done as both invasive species appear to be spreading at different rates within these regions [12], which may show dissimilar spatio-temporal autocorrelation patterns.

### Spatial data

One objective of this study was to use widely available spatial data in a statistical model to describe the current distribution and predict sites with suitable conditions for future invasion. Invasive species are operationally defined as requiring human involvement [29], usually via introduction pathways (e.g., shipping terminals) or dispersal corridors (e.g., roads), and all species have environmental tolerances outside which they cannot survive. Therefore, we included variables under the categories of anthropogenic and environmental to broadly describe the “habitat” of each county. Predictor datasets were obtained from the National Atlas of the United States (United States Department of the Interior 2003) and ESRI (ESRI Data & Maps 2004, Redlands, CA, USA). Datasets in the anthropogenic category include 2003 human population, length of railroad tracks (km), length of major highways (km), number of transportation terminals (airport, seaport, bus, and rail), proportion of county area classified as agriculture, proportion of county area classified as urban land use, and proportion of population in urban areas. Datasets in the environmental category include mean annual precipitation (mm), minimum temperature (C), mean elevation (m), presence of river within or bordering county, and county area (km^2^). A single value for each predictor was determined for each county, and collated into a single database.

### Logistic regression analysis

The herbarium record database for each species was appended to the “habitat” variable database, which was exported in tabular format for statistical analyses. The dependent variable was binomially distributed–herbarium record = 1 and lack of record = 0. All predictor variables were examined for normality and transformed as necessary. County population, area, mean elevation, and mean precipitation were log_10_ transformed to achieve homoskedasticity, and a constant (10) was added to minimum temperature means to make all values positive.

As we had no *a priori* hypotheses regarding which predictor variables would provide more robust estimates of habitat suitability, we randomly divided the data for each species into a training dataset to fit the model (using 2/3 of the data) and a test dataset to check its accuracy (using the remaining 1/3 of the data). To determine the minimum number of variables describing the geographic distribution of each species we ran a stepwise-mixed logistic regression analysis, which combines both forward and backward variable selection, on the training dataset (JMP v5.1), with county area as a covariate and a tolerance threshold for entering and keeping a variable in the model at *P*≤0.10. Diagnostics for model fit (Hosmer & Lemeshow) and accuracy (difference in deviance by estimated probability plot) were checked for each species. Standardized regression coefficients were calculated for each predictor in the model to allow comparison among variables with different units [30]. The logistic model with the best performance was then checked for accuracy with the test dataset.
